# Supplementary material for: Host migration and environmental temperature influence avian haemosporidians prevalence: a molecular survey in a Brazilian Atlantic rainforest
Source: PeerJ. 2021 Jun 22;9:e11555. doi: 10.7717/peerj.11555 (PMC8231341; doi:10.7717/peerj.11555)
Supplement: Supplemental Information 1 — The infection status, and parasites’ genus and lineages, when identified, are provided. The sample size and number of infections detected in each bird species, and the number of birds infected by a parasite lineage are denoted in parentheses. Because not all diagnosed infections were sequenced, the number of birds in the “Infection Status” column may not be equivalent to the number in the “Lineage” column. [file peerj-09-11555-s001.pdf]

SUPPLEMENTARY MATERIALS

Host migration and environmental temperature influence avian haemosporidian prevalence: a molecular survey in a Brazilian Atlantic Rainforest

Raquel A. Rodrigues[1], Gabriel M. F. Felix[2], Mauro Pichorim[3], Patrícia A. Moreira[4], Érika M. Braga[1*]

Table S1: Migratory birds captured in a bird community in Barreira do Inferno Rocket Launch Center of the Brazilian Air Force, Parnamirim, State of Rio Grande do Norte, Brazil. The infection status, and parasites' genus and lineages, when identified, are provided. The sample size and number of infections detected in each bird species, and the number of birds infected by a parasite lineage are denoted in parentheses. Because not all diagnosed infections were sequenced, the number of birds in the "Infection Status" column may not be equivalent to the number in the "Lineage" column.

Host Species		Infection Status	Parasite Genus	Lineage
Scientific name	Common name			
Coccyzus melacoryphus (2)	Dark-billed Cuckoo	Non infected	-	-
Columbina picui (1)	Picui dove	Non infected	-	-
Cyanerpes cyaneus (1)	Red-legged Honeycreeper	Infected (1)	Non sequenced	-
Elaenia chilensis (244)	Chilean Elaenia	Infected (40)	*Plasmodium* and *Haemoproteus*	*Plasmodium* BAFLA03 (1), BAFLA04 (1), PADOM09 (1), PADOM17 (1), TURNUD02,<br><br>*Haemoproteus* ELALB01(1), UN203 (1)
Elaenia chiriquensis (2)	Lesser Elaenia	Non infected	-	-
Elaenia cristata (96)	Plain-crested Elaenia	Infected (12)	Non sequenced	-

Elaenia spectabilis (9)	Large Elaenia	Infected (2)	*Plasmodium*	BAFLA04 (1)
Empidonomus varius (1)	Variegated Flycatcher	Non infected	-	-
Myiarchus tyrannulus (4)	Brown-crested Flycatcher	Infected (1)	*Haemoproteus*	ELALB01 (1)
Myiodynastes maculatus (1)	Northern Streaked Flycatcher	Non infected	-	-
Schistochlamys ruficapillus (9)	Cinnamon Tanager	Infected (2)	Non sequenced	-
Tangara sayaca (7)	Sayaca Tanager	Non infected	-	-
Turdus amaurochalinus (156)	Creamy-bellied Thrush	Infected (20)	*Plasmodium*	U12 (1), TUAMA01 (1), DENPET03 (1)
Turdus flavipes (11)	Yellow-legged Thrush	Infected (3)	*Plasmodium* and *Haemoproteus*	*Plasmodium* DENPET03 (1), PADOM09 (1) *Haemoproteus* TARUF02 (1)
Vireo chivi (10)	Red-eyed Vireo	Infected (5)	*Haemoproteus*	VIREO02 (1)
