# Supplementary material for: Host migration and environmental temperature influence avian haemosporidians prevalence: a molecular survey in a Brazilian Atlantic rainforest
Source: PeerJ. 2021 Jun 22;9:e11555. doi: 10.7717/peerj.11555 (PMC8231341; doi:10.7717/peerj.11555)
Supplement: Supplemental Information 2 — The full random structure was maintained in all models. The Plas.Glmm1 model starts with all main effects (fixed factors), which were sequentially removed. In each step we removed the main effect that explained the small amount of deviance (indicated on the fixedfactor column). The procedure was repeated until no effect could be removed without causing significant loss of model fit (indicated by the p value column). In this case, this happened only when temperature was removed from the model. [file peerj-09-11555-s002.pdf]

SUPPLEMENTARY MATERIALS

Host migration and environmental temperature influence avian haemosporidian prevalence: a molecular survey in a Brazilian Atlantic Rainforest

Raquel A. Rodrigues[1], Gabriel M. F. Felix[2], Mauro Pichorim[3], Patrícia A. Moreira[4], Érika M. Braga[1*]

Table S2. Backward selection of the main effects influencing the probability of infection by *Plasmodium* sp. The full random structure was maintained in all models. The Plas.Glmm1 model starts with all main effects (fixed factors), which were sequentially removed. In each step we removed the main effect that explained the small amount of deviance (indicated on the fixed factor column). The procedure was repeated until no effect could be removed without causing significant loss of model fit (indicated by the p value column). In this case, this happened only when temperature was removed from the model.

Model	Fixed Factors	Deviance	Chisq	DF	P
**Plas.Glmm1**	Full[ɫ]	243.03			
**Plas.Glmm2**	- Age	243.03	0.002	1	0.959
**Plas.Glmm3**	- Preciptation	243.09	0.063	1	0.800
**Plas.Glmm4**	- Migratory status	244.72	1.621	1	0.202
**Plas.Glmm5**	- Nest shape	245.80	1.080	1	0.298
**Plas.Glmm6**	- Body mass	248.18	2.378	1	0.122
**Plas.Glmm7**	- Breeding period	249.43	1.251	2	0.534
**Plas.Glmm8 (null)**	- Temperature	257.62	8.195	1	0.004**

[ɫ]Full model: *Plasmodium* sp. Infection status ~ Intercept + Age + Precipitation + Migratory Status + Nest Shape + Body mass + Breeding Period + Temperature + (Intercept|Season/Month) + (Intercept|Order/Family/Genus/Species), family = binomial (link = “logit”).
