# Supplementary material for: Host migration and environmental temperature influence avian haemosporidians prevalence: a molecular survey in a Brazilian Atlantic rainforest
Source: PeerJ. 2021 Jun 22;9:e11555. doi: 10.7717/peerj.11555 (PMC8231341; doi:10.7717/peerj.11555)
Supplement: Supplemental Information 3 — The full random structure was maintained in all models.The Plas.Glmm1 model starts with all main effects (fixed factors), which were sequentially removed. In each step we removed the main effect that explained the small amount of deviance (indicated on the Fixed Factors column). The procedure was repeated until no effect could be removed without causing significant loss of model fit (indicated by the p value column). In this case, this happened only when migratory status was removed from the model, although theimprovement in deviance provided by Body mass and Breeding period is marginally significant, indicating a possible role of these factors in the probability of infection by Haemoproteus (Parahaemoproteus) sp. [file peerj-09-11555-s003.pdf]

SUPPLEMENTARY MATERIALS

Host migration and environmental temperature influence avian haemosporidian prevalence: a molecular survey in a Brazilian Atlantic Rainforest

Raquel A. Rodrigues[1], Gabriel M. F. Felix[2], Mauro Pichorim[3], Patrícia A. Moreira[4], Érika M. Braga[1*]

Table S3. Backward selection of the main effects influencing the probability of infection by *Haemoproteus* (*Parahaemoproteus*) sp. The full random structure was maintained in all models. The Plas.Glmm1 model starts with all main effects (fixed factors), which were sequentially removed. In each step we removed the main effect that explained the small amount of deviance (indicated on the Fixed Factors column). The procedure was repeated until no effect could be removed without causing significant loss of model fit (indicated by the p value column). In this case, this happened only when migratory status was removed from the model, although the improvement in deviance provided by Body mass and Breeding period is marginally significant, indicating a possible role of these factors in the probability of infection by *Haemoproteus* (*Parahaemoproteus*) sp.

Model	Fixed Factors	Deviance	Chisq	DF	P
**Para.Glmm1**	Full[ɪ]	180.31			
**Para.Glmm2**	- Precipitation	180.44	0.133	1	0.715
**Para.Glmm3**	- Nest shape	181.36	0.916	1	0.338
**Para.Glmm4**	- Age	182.34	0.979	1	0.322
**Para.Glmm5**	- Temperature	183.07	0.735	1	0.391
**Para.Glmm6**	- Body mass	185.83	2.754	1	0.096
**Para.Glmm7**	- Breeding period	190.34	4.643	2	0.098
**Para.Glmm8 (null)**	- Migratory status	200.34	9.867	1	0.001**

[ɪ]Full model: *Parahaemoproteus sp.* Infection status ~ Intercept + Age + Precipitation + Migratory Status + Nest Shape + Body mass + Breeding Period + Temperature + (Intercept|Season/Month) + (Intercept|Order/Family/Genus/Species), family = binomial(link = “logit”).
