# Supplementary material for: Host migration and environmental temperature influence avian haemosporidians prevalence: a molecular survey in a Brazilian Atlantic rainforest
Source: PeerJ. 2021 Jun 22;9:e11555. doi: 10.7717/peerj.11555 (PMC8231341; doi:10.7717/peerj.11555)
Supplement: Supplemental Information 4 — The factors were considered as two dimensions of the random structure of the models built to analyze the factors predicting the probability of infection by Plasmodium and Haemoproteus in a bird community in Barreira do Inferno Rocket Launch Center of the Brazilian Air Force, Parnamirim, State of Rio Grande do Norte, Brazil. [file peerj-09-11555-s004.pdf]

SUPPLEMENTARY MATERIALS

Host migration and environmental temperature influence avian haemosporidian prevalence: a molecular survey in a Brazilian Atlantic Rainforest

Raquel A. Rodrigues[1], Gabriel M. F. Felix[2], Mauro Pichorim[3], Patrícia A. Moreira[4], Érika M. Braga[1*]

Table S4. Adjusted intra-class correlation (ICC_{adj}) of temporal and taxonomic factors. The factors were considered as two dimensions of the random structure of the models built to analyze the factors predicting the probability of infection by *Plasmodium* and *Haemoproteus* in a bird community in Barreira do Inferno Rocket Launch Center of the Brazilian Air Force, Parnamirim, State of Rio Grande do Norte, Brazil.

Dimension	Random Factor	ICC_{adj} (*Plasmodium* sp.)	ICC_{adj} (*Parahaemoproteus* sp.)
**Temporal**	Season	0	0
	Month:Season	0	0
**Taxonomic**	Family	0	0
	Genus:Family	0	0
	Species:Genus:Family	0.1728	0
